# A Preliminary Characterisation of Cognition and Social Cognition in Spinocerebellar Ataxia Types 2, 1, and 7

**DOI:** 10.3233/BEN-2010-0270

**Published:** 2010-08-16

**Authors:** N. Sokolovsky, A. Cook, H. Hunt, P. Giunti, L. Cipolotti

**Affiliations:** ^1^Department of NeuropsychologyNational Hospital for Neurology and NeurosurgeryQueen SquareLondonUK; ^2^Department of Molecular NeuroscienceInstitute of NeurologyUCLQueen SquareLondonUK; ^3^Dipartimento Di PsicologiaUniversità Degli Studi Di PalermoPalermoItaly

**Keywords:** Spinocerebellar ataxia (SCA), cognition, theory of mind, emotion

## Abstract

Over the last decade, studies have implicated the cerebellum not only in motor functioning, but also in cognition and social cognition. Although some aspects of cognition have been explored in the five most common forms of Spinocerebellar Ataxia (SCA), social cognition in these patients has rarely been examined. The present study provides a preliminary characterisation of the severity of cognitive and social cognitive impairments in patients with SCA2, SCA1 and SCA7 using an identical battery to the one previously used in SCA3 and SCA6 patients for comparison. The cognitive profiles of SCA1 and SCA7 patients were comparable to that of SCA6 patients; SCA1 patients had relatively intact profiles, while SCA7 patients demonstrated only some selective deficits. In contrast, SCA2 patients showed the greatest impairments, similarly to SCA3 patients. On tests of social cognition, SCA2 and SCA7 patients were impaired on a task of emotion attribution, whereas one SCA1 patient had a Theory of Mind deficit, which has also been documented in SCA3 and SCA6. We provide preliminary evidence that the neuropsychological profiles of SCA patients correspond well with the severity of pathological and clinical features. Moreover, these patients may also have social cognition impairments. Overall, we suggest that there is a degree of heterogeneity in the types of cognitive and social cognitive impairments in SCA patients.

## 1. Introduction

The spinocerebellar ataxias (SCAs) are a group of rare (prevalence rate of 1–4 in 100,000 [55]) neurodegenerative disorders of autosomal dominant inheritance, which share the unifying characteristic of progressive ataxia resulting from degeneration of the cerebellum and its connections. Although the prevalence of each subtype varies according to ethnicity and geography, SCA1, SCA2, SCA3, SCA6, and SCA7 are universally the most common types [55]. SCA2, SCA1, and SCA7 – the focus of this study – are caused by expansions of CAG repeats at chromosomes 12q23-24.1, 6p23, and 3p12-p21.1, respectively [25,34,64].

The SCAs are heterogeneous in their clinical manifestations, showing huge overlap across subtypes and great variability within subtypes [78]. However, the phenotypes of patients of the same subtype may be more homogenous than previously believed, if taking into account the patient's age of onset and disease duration [81]. SCA2, SCA1 and SCA7 are all characterised by cerebellar ataxia, pyramidal and extrapyramidal signs, oculomotor abnormalities and amyotrophy, which may develop at different times as the disease progresses (e.g. [17,18,29]). Despite these overlaps, the clinical profiles of these three patient groups do differ in some aspects. SCA2 patients show more

[1] P.G. and L.C. are the senior authors.
*Corresponding author: Prof. Lisa Cipolotti, Department of Neuropsychology, Box 37, National Hospital for Neurology and Neurosurgery, Queen Square, WC1N 3BG, London, UK. Tel.: +91 020 7829 8793; Fax: +91 080 7813 2516; E-mail: l.cipolotti@ion.ucl.ac.uk.

ISSN 0953-4180/10/$27.50 © 2010 – IOS Press and the authors. All rights reserved extrapyramidal symptoms and more pronounced saccadic slowing, hyporeflexia and tremor, as well as frequent sensorimotor neuropathy [35,78]. The clinical deficits of SCA1 patients are somewhat less severe, and sensorimotor neuropathy is seen in only 50% of cases [28,69,97]. Patients with SCA7 present with no sensorimotor neuropathy and are distinguishable by the presence of pigmentary maculopathy, but their clinical profile varies greatly depending on the number of CAG repeats [36,41,52,69].

These clinical profiles coincide well with the pathological appearance of the three SCA types. SCA2 is associated with the most widespread atrophy, which includes olivopontocerebellar circuits, supratentorial regions, the thalamus, parts of the cerebral cortex, and the upper spinal cord [9,26,27,74,85]. SCA1 is characterised by less pronounced olivopontocerebellar atrophy and some degeneration of the upper spinal cord [40, 50,81]. The pathology of SCA7 also revolves around the olivopontocerebellar circuits, although it has been suggested that pontine atrophy in these patients may precede cerebellar atrophy [3,4].

Although it was originally believed that the cerebellum is involved only in motor functions, numerous neuropsychological studies have now implicated its role in cognition. In patients with congenital or acquired cerebellar conditions, the cognitive impairments most consistently found are executive dysfunction, visuospatial deficits and language difficulties [2,39,53, 87]. Schmahmann and colleagues [76,77] termed this constellation of cognitive impairments the Cerebellar Cognitive Affective Syndrome (CCAS), and suggested that it may be the result of disruptions to the cerebrocerebellar circuitry. Given the common underlying cerebellar dysfunction that is characteristic of all SCA types, finding such cognitive impairments in SCA patients would provide further evidence to support the cerebellum's role in cognition.

To date, studies evaluating the cognitive functioning of patients with the most common types of SCA have concentrated mostly on SCA1, SCA2, and SCA3, and some on SCA6. Studies of the first three SCA types have consistently documented impairments in frontal executive functions, verbal memory, and attention [13–15,31,48,51,58,85]. The few papers that have looked at SCA6 patients recorded some deficits on tasks of executive function and visual memory [32,37,47,86]. However, to the best of our knowledge, there have been no studies examining the cognitive profile of patients with SCA7. Furthermore, there have been no direct comparisons of these five most prevalent types of SCA using a common, comprehensive neuropsychological battery. In this study, we examined patients with SCA2, SCA1 and the less-explored SCA7, using the same detailed neuropsychological battery that we have used previously in patients with SCA3 and SCA6 (see [32]).

In addition to its contribution to cognition, there is also evidence to suggest that the cerebellum may be involved in social cognition. Indeed, the CCAS also referred to behavioural abnormalities and personality changes observed in patients. This finding is confirmed by other studies of cerebellar patients with behavioural disturbances, personality change, social isolation, and marked deficits in understanding social cues [20,67,71, 87].

The findings of bidirectional pathways linking the cerebellum to limbic structures have given rise to the suggestion that the former may play a role specifically in emotional processing [42–44]. Using activation likelihood estimate meta-analysis, Stoodley and Schmahmann [84] confirmed that emotional processing consistently activated cerebellar lobules VI and VII, and Crus I. Studies of cerebellar patients suggest its involvement in the subjective experience of emotion, with evidence of flat affect, impaired affect regulation, and pathological laughter or crying [65,77]. These patients also show reduced activity in the limbic system in response to fear-inducing stimuli [89]. Moreover, cerebellar stimulation in healthy controls can lead to increased negative mood in response to unpleasant stimuli [80]. Notably, the cerebellum may also be associated with the perception of emotion in others, as shown in healthy participants [45,79].

Several studies have also suggested that the cerebellum may be involved in a different aspect of social cognition, namely the ability to attribute mental states to others, which is known as Theory of Mind (ToM). Van Harskamp et al. [90] tested patients with superficial siderosis, a condition involving predominantly the cerebellum, and found a specific ToM impairment, despite normal performance on emotion attribution and social judgement tasks. Abel et al. [1] provided more localised evidence for this suggestion by revealing ToM deficits in patients suffering from isolated cerebellar degenerative disease. Moreover, imaging studies of healthy controls have also demonstrated strong cerebellar activation during ToM tasks [10,16]. Interestingly, ToM impairment is also one of the hallmarks of autism, which has been associated with cerebellar abnormalities, most commonly atrophy of vermal lobules VI and VII [5,23,73,93]. The importance of the cerebellum's role in this syndrome is highlighted by the

Table 1
Demographic and clinical characteristics of SCA2, SCA1 and SCA7 patients

| Pt No. | SCA type | Occupation | Age (yrs) | Sex | Years of education | NART premorbid FIQ | Age at disease onset | Disease duration (yrs) | Truncal ataxia | Upper limb ataxia | Dysarthria | Comorbid conditions and/or medication |
|---|---|---|---|---|---|---|---|---|---|---|---|---|
| 1 | SCA2 | Teacher | 55 | F | 13 | 118 | 44 | 11 | Mild | Mild | Mild | Essential hypertension<br>– Enalapril<br>– Vitamin B12 |
| 2 | SCA2 | Pharmacist | 50 | M | 16 | 103 | 37 | 13 | Mild | Mild | Mild | None |
| 3 | SCA2 | Customer Services Agent | 49 | F | 10 | 96 | 44 | 5 | Moderate | Mild | Mild | None<br>– Paracetamol |
| 4 | SCA1 | Counter technician | 36 | M | 11 | 92 | 31 | 5 | Mild | Mild | Mild | None |
| 5 | SCA1 | Taxi driver | 36 | M | 15 | 112 | 30 | 6 | Mild | Mild | Mild | Previous alcoholism<br>– Lansoprazole |
| 6 | SCA7 | Secondary school teacher | 50 | F | 18 | 116 | 38 | 12 | Moderate | Mild | Mild | None<br>– Co-codamol |
| 7 | SCA7 | Civil servant | 39 | F | 17 | 98 | 34 | 5 | Mild | Mild | Mild | None |
| 8 | SCA7 | Social services project manager | 38 | M | 17 | 103 | 29 | 9 | Mild | Mild | None | None |

yrs = years; NART = National Adult Reading Test; FIQ = Full Scale Intelligence Quotient; Pt = Patient; No. = Number.

finding that cerebellar white matter volume and vermis size can distinguish 95% of toddlers with autism [22].

In line with the above suggested role of the cerebellum, both SCA3 and SCA6 patients in our previous study showed some impairment on social cognition tests [32]. Both groups performed poorly on a ToM task in which they were asked to justify the behaviour of characters in short stories. They provided fewer mental state and more physical state justifications than controls. However, their performance on a task of emotion attribution, in which they had to decide how the character of a short story was feeling, was comparable to that of controls. To date, this has been the only study to explore emotion attribution and ToM in patients suffering from the five most common forms of SCA.

In this preliminary study, we extend our previous findings by examining patients who suffer from the other three most prevalent SCA types – SCA2, SCA1 and SCA7 – using an identical battery of cognitive, emotional attribution and ToM tasks. To the best of our knowledge, this is the first study to examine social cognition in these three SCA types, and the first to evaluate general cognition in SCA7. The aim of the study was to provide a preliminary assessment of the severity of cognitive and social impairments in these five neurodegenerative cerebellar disorders, using a comprehensive and identical battery, and thus determine which functions may critically depend upon the cerebellum.

## 2. Methods

### 2.1. Subjects

Eight genetically confirmed SCA2, SCA1 and SCA7 patients (three patients with SCA2, two with SCA1, and three with SCA7) were assessed in the Neuropsychology Department of the National Hospital for Neurology and Neurosurgery. Age, symptom duration, age at disease onset, demographic characteristics and salient clinical details about each patient in the study are displayed in Table 1. With one exception, the occupations pursued by members of the three groups were all white-collar jobs. The mean age of the three patient groups differed significantly only between the SCA1 and SCA2 groups ($t(3) = -6.4$, $p = 0.008$), with an older mean age for SCA2 patients. The mean age at disease onset also differed significantly between these two groups ($t(3) = -3.68$, $p = 0.035$), with later onset in SCA2 patients, but no such differences were found with SCA7 patients. All three groups were matched in terms of sex distribution [$\chi^2$(2, N = 8) = 2.33, $p$ = 0.31], years of formal education (SCA1 and SCA2, $p$ = 1.0; SCA1 and SCA7, p=0.27; SCA2 and SCA7, $p$ = 0.07), estimated premorbid IQ (SCA1 and SCA2, $p$ = 0.77; SCA1 and SCA7, $p$ = 0.74; SCA2 and SCA7, $p$ = 1.0), and disease duration (SCA1 and SCA2, $p$ = 0.28; SCA1 and SCA7, $p$ = 0.32; SCA2 and SCA7, $p$ = 0.77).

The degree of physical disability suffered by each group was similar. When mild, moderate and severe impairment in each of the three clinical domains (truncal ataxia, upper limb ataxia and dysarthria) were given nominal values of 1, 2 and 3 respectively, there was no difference between the mean composite disability scores of the three patient groups [$\chi^2$(2, N = 8) = 0.48, $p$ = 0.79]. It is also apparent from Table 1 that one patient with SCA1, two with SCA2, and one with SCA7 were taking regular medication, though only one medication (Co-codamol) has a potential harmful effect on cognitive function, specifically short-term memory. Detailed interviews were conducted by a neurologist to ascertain whether any patients had psychiatric illnesses. None of the patients showed any major psychiatric disorders, in particular no anxiety or depressive traits were noted. None of the patients were taking antidepressants or any other psychopharmacological drugs (see Table 1).

Four of the eight patients underwent MRI/CT scans at their regional hospital (Patients 1, 3, 6 and 7). For these patients, such neuroradiological investigations excluded the presence of additional vascular, space-occupying, inflammatory or neurodegenerative lesions. Notably, no white matter pathology was found in the only SCA2 patient who was hypertensive.

The study was approved by the joint research ethics committee of the National Hospital for Neurology and Neurosurgery and the Institute of Neurology and performed in accordance with the ethical standards prescribed by the 1964 Declaration of Helsinki. All participants gave informed consent before participating.

### *2.2. Cognitive and social cognitive evaluation*

Participants were assessed using a comprehensive battery of neuropsychological tests, known to be sensitive to subtle cognitive deficits and for which age-standardised test scores were available. In addition, an extended battery of social cognition tests was administered. Where published norms were not available, the patients' performance was compared with that of a group of 22 age-matched healthy controls.

### *2.3. General neuropsychological battery*

The cognitive battery assessed the following domains: Current intellectual functioning (Wechsler Adult Intelligence Scale – Revised [94]); Premorbid intellectual functioning (National Adult Reading Test, NART [62]); Verbal and visual recognition memory (Recognition Memory Test [91]); Verbal and visual recall memory (Wechsler Memory Scale – Word Lists and Visual Reproduction [95]); Nominal functions (Graded Naming Test [56]); Calculation (Graded Difficulty Arithmetic Test [46]); Perceptual functions (Visual Object and Space Perception battery – Incomplete Letters [92]); Attention (Test of Everyday Attention – Elevator Counting Task and Elevator Counting with Distraction [72]); and executive functions (Verbal Fluency [82], Stroop Test [88], Hayling Sentence Completion Test [11], Modified Card Sorting Test [61]).

Four derived scores, based on normative data collected on a normal sample of comparable age, were calculated as follows:

1. The intellectual functioning score was the difference between the NART and the Full Scale IQ. In order to maintain consistency with our previous study of SCA3 and SCA6 patients [32], a difference greater than 15 was taken as evidence of intellectual decline. Notably, a difference of 15 is often used as a standard measure for evaluating intellectual decline [96].
2. Recognition and recall memory, naming, calculation, one speed and attention test (Elevator Counting with Distraction) and two executive test (Verbal Fluency and Stroop) scores were derived by converting standardised test performance into percentile scores. Scores at, or below the 5th percentile indicated impairment.
3. Raw scores on the Hayling Sentence Completion Test were converted into scaled scores. According to published norms, a scaled score of three or below indicated impairment.
4. Perception scores at or below the 5% cut-off indicated perceptual impairment.

### *2.4. Social cognition battery*

This battery consisted of two tests originally devised by Blair and Cipolotti [7] and adapted by Van Harskamp et al. [90]. Examples of each of the tests are provided in the Appendix.

### 2.5. Emotion attribution

In this test, the participant was presented with 75 short stories describing an emotional situation and was required to provide a one-word description of how the main character might feel in that situation. The sentences were designed to elicit happiness, sadness, fear, anger or embarrassment. The test was scored according to the number of correct attributions made for each emotion category, i.e. participants received a score out of 15.

### 2.6. Theory of mind

In this test, the participant was presented with 14 stories describing naturalistic social situations and was asked to interpret and justify the behaviour of the main character. Three scores were recorded. The first indexed comprehension of the situation (maximum 14). The remaining two scores referred to the justification provided during interpretation of the story character's behaviour, i.e. reference to either the character's mental state or physical information (see Appendix for examples of these two types of responses). Of the stories correctly understood, the total number of stories justified using mental state information and the total number of stories justified with reference to physical information were recorded.

## 3. Results

### 3.1. Performance on the general neuropsychological battery

In all three patient groups, perceptual and calculation functions were found to be preserved. Nominal impairments were found only in one patient with SCA2. Verbal and visual recognition and recall memory were preserved in all patients, with the exception of one patient with SCA7 who showed impaired visual recognition. Attentional deficits were only noted in SCA1 and SCA2, and were more common in the latter.

Intellectual impairments were found in all three patient groups. Interestingly, these consisted of a selective impairment of performance IQ, which assesses non-verbal abstract reasoning functions. We analysed the errors the patients made on two of the timed non-verbal subtests of the WAIS-R that required motor control (Picture Arrangement and Block Design). The patients' errors were not due to an inability to finish an item within the time limit. Moreover, an analysis of the means and standard deviations of the patients' age-scaled scores on these two non-verbal subtests (Picture Arrangement, $\overline{x} = 8$, sd $= 2$; Block Design, $\overline{x} = 9$, sd $= 2.14$) revealed that these did not differ from their scores on another non-verbal subtest, the Picture Completion subtest, which does not require good motor control ($\overline{x} = 9.13$, sd $= 2.76$). These analyses indicate that the patients' poor performance on the non-verbal part of the WAIS-R is unlikely to be solely the result of cognitive slowness and/or motor difficulties associated with ataxia.

Frontal executive impairments were noted in SCA2 and SCA7. In both patient groups, impairments were noted in three of the four tasks used to evaluate executive functioning. Interestingly, neither of the two groups showed any impairment on the Modified Card Sorting Test. Remarkably, frontal executive deficits were absent in SCA1.

Table 2a reports the cognitive performance of patients with each of the three different types of SCA as a group. Table 2b details the individual performance of the patients. In both tables, all the test scores are reported as raw scores, the only exceptions being the IQ scores and the Hayling test scores.

### 3.2. Performance on the social cognition battery

Tables 3 and 4 show the scores achieved by each of the patients and the mean scores of the controls on the Emotion Attribution and Theory of Mind tasks of the social cognition test battery. The pattern of results for each subtest will be discussed separately. Given the small sample sizes in both the patient and control groups, the performance of each patient was compared with that of the control group using the modified t-test [24].

### 3.3. Emotion attribution task (*Table 3*)

None of the patients with SCA1 differed significantly from controls in the number of correct attributions of any of the five emotions.

In the two SCA7 patients who were administered this task, impairments were selective for certain emotions. Emotional attributions in one patient were reduced only for the emotions of happiness and sadness ($p < 0.001$ and $p = 0.009$, respectively), whereas for another patient the impairment was only found for attributions of embarrassment ($p = 0.026$).

Table 2a
Summary of group performance on cognitive tests for SCA2, SCA1 and SCA7 patients (Means and SDs of raw scores$^{+}$)

| Neuropsychological tests | SCA2 ($n = 3$) | | | SCA1 ($n = 2$) | | | SCA7 ($n = 3$) | | |
|---|---|---|---|---|---|---|---|---|---|
| | Mean (sd) | Range | Number impaired | Mean (sd) | Range | Number impaired | Mean (sd) | Range | Number impaired |
| **Perception VOSP** | | | | | | | | | |
| Incomplete Letters | 19.33(1.15) | 18–20 | 0 | 19.5(0.71) | 19.20 | 0 | 19.33(1.15) | 18–20 | 0 |
| **Calculation** | | | | | | | | | |
| GDAT | 13.67(5.86) | 7–18 | 0 | 20.5(4.95) | 17–24 | 0 | 12.67(8.33) | 6–22 | 0 |
| **Nominal Functions** | | | | | | | | | |
| GNT | 17.67(6.11) | 11–23 | **1** | 20.5(0.71) | 20–21 | 0 | 21.33(6.11) | 16–28 | 0 |
| **Recognition Memory** | | | | | | | | | |
| RMW | 45.67(4.93) | 40–49 | 0 | 41(1.41) | 40–42 | 0 | 49.67(0.58) | 49–50 | 0 |
| RMF | 39.33(0.58) | 39–40 | 0 | 41.5(0.71) | 41–42 | 0 | 39.67(6.11) | 33–45 | **1** |
| **Recall Memory** | | | | | | | | | |
| WMS-III Word Lists Delayed Recall/12 | 4.33(1.53) | 3–6 | 0 | 8.5(0.71) | 8–9 | 0 | 8.33(1.53) | 7–10 | 0 |
| WMS-III Visual Reproduction Delayed Recall/104 | 54.67(13.61) | 44–70 | 0 | 64.5(28.99) | 44–85 | 0 | 39(15.59) | 30–57 | 0 |
| **Attention (TEA)** | | | | | | | | | |
| Elevator Counting /7 | 7(0) | 7–7 | 0 | 7(0) | 7–7 | 0** | 6.67(0.58) | 6–7 | 0 |
| Elevator Counting with Distraction | 4.67(2.52) | 2–7 | **2** | 6(5.66) | 2–10 | **1** | 8.33(1.53) | 7–10 | 0 |
| **Intellectual Functioning** | | | | | | | | | |
| WAIS-R | | | | | | | | | |
| Full Scale IQ[1] | 93(5.2) | 87–96 | **1** | 97(4.24) | 94–100 | **1** | 99.33(20.79) | 84–123 | 0 |
| Verbal IQ[1] | 99.67(5.77) | 93–103 | 0 | 101(2.83) | 99–103 | 0 | 102.67(17.01) | 90–122 | 0 |
| Performance IQ[1] | 86.33(5.03) | 81–91 | **3** | 93(12.73) | 84–102 | **1** | 94(21.17) | 78–118 | **2** |
| NART Full Scale IQ[1] | 105.67(11.24) | 96–118 | – | 102(14.14) | 92–112 | 0 | 105.67(9.29) | 98–116 | – |
| **Executive Functions** | | | | | | | | | |
| Verbal Fluency FAS | 34.67(12.74) | 20–43 | **1** | 36.5(6.36) | 32–41 | 0 | 31.33(8.39) | 26–41 | **1** |
| Hayling Test[2] | 14(4.58) | 10–19 | **1** | 18(0) | 18–18 | 0 | 13(4.58) | 9–18 | **2** |
| Stroop C-W Test | 70.33(9.71) | 62–81 | **1** | 110(2.83) | 108–112 | 0 | 41.5(10.61) | 34–49 | **2*** |
| MCST (categories) | 6(0) | 6–6 | 0 | 6(0) | 6–6 | 0 | 6(0) | 6–6 | 0 |

$^{+}$Except for IQ and Hayling scores; [1]Results presented as IQ scores; [2]Results presented as total scaled scores.
*Only two SCA7 patients were administered this test; **Only one SCA1 patient was administered this test.
Abbreviation key: VOSP = Visual Object and Space Perception battery; GDAT = Graded Difficulty Arithmetic Test; GNT = Graded Naming Test; RMW = Recognition Memory test for Words; RMF = Recognition Memory test for Faces; WMS-III = Wechsler Memory Scale 3rd edition; TEA = Test of Everyday Attention; WAIS-R = Wechsler Adult Intelligence Scale-Revised; IQ = Intelligence Quotient; NART = National Adult Reading Test; FAS = Verbal Fluency FAS; Hayling = Hayling Sentence Completion test; C-W = Stroop Colour-Word task; MCST = Modified Card Sorting Test; sd=standard deviation; No. = number.

In contrast, a much more pervasive impairment in emotional attribution was found in the SCA2 patient group. One patient provided significantly fewer correct attributions than controls for all five emotions (happiness: $p = 0.004$; sadness: $p = 0.002$; fear: $p = 0.002$; anger: $p = 0.011$; embarrassment: $p = 0.026$). The other two patients were impaired in the attribution of the emotions of fear, anger and sadness, respectively ($p = 0.026$ and $p = 0.011$, $p = 0.034$).

### 3.4. *Theory of mind task* (*Table 4*)

There were no significant differences between the patients and the controls on the number of stories correctly comprehended, with the exception of one SCA2 patient who misunderstood one story ($p = 0.004$). However, although this patient misunderstood the story, they had no difficulty in providing a mental state justification for the behaviour of its main character. One of the two patients with SCA1 was significantly different to controls in the number of mental and physical state justifications provided. Specifically, this patient provided fewer mental state and more physical state justifications than controls (for mental justifications: $p < 0.001$; for physical justifications: $p < 0.001$). There were no significant differences between any of the other patients and controls in these justifications. These results suggest a potential impairment in the ability of SCA1 patients to judge the internal mental states of others.

Table 2b
Summary of individual performance on cognitive tests for patients with SCA2, SCA1 and SCA7

| Neuropsychological tests | Patient number | SCA2 patients 1 | 2 | 3 | SCA1 patients 4 | 5 | SCA7 patients 6 | 7 | 8 |
|---|---|---|---|---|---|---|---|---|---|
| **Perception VOSP** | | | | | | | | | |
| Incomplete Letters | | > cut-off | > cut-off | > cut-off | > cut-off | > cut-off | > cut-off | > cut-off | > cut-off |
| **Calculation** | | | | | | | | | |
| GDAT* | | 25th | > 75th | 75th | >75th | > 75th | > 75th | 25th | 25–50th |
| **Nominal Functions** | | | | | | | | | |
| GNT* | | 75th | <5th | 25–50th | 50–75th | 50th | > 95th | 10–25th | 50th |
| **Recognition Memory** | | | | | | | | | |
| RMW* | | > 75th | 10th | > 75th | 10–25th | 5–10th | 90th | > 75th | > 75th |
| RMF* | | 25th | 10th | 10–25th | 25–50th | 25th | 25th | < 5th | 50–75th |
| **Recall Memory** | | | | | | | | | |
| WMS-III Word Lists | | | | | | | | | |
| Delayed Recall* | | 41–59th | 19–28th | 41–59th | 72–81rst | 82–89th | 72–81rst | 41–59th | 90–94th |
| WMS-III Visual Reproduction | | | | | | | | | |
| Delayed Recall* | | 41–59th | 72–81rst | 29–40th | 82–89th | 19–28th | 11–18th | 11–18th | 29–40th |
| **Attention (TEA)** | | | | | | | | | |
| Elevator Counting | | Normal | Normal | Normal | Normal | nt | Normal | Normal | Doubtful |
| Elevator Counting with | | | | | | | | | |
| Distraction* | | 10–25th | 10th | 5th | 75th | 5th | 75th | 25th | 25–50th |
| **Intellectual Functioning** | | | | | | | | | |
| WAIS-R | | | | | | | | | |
| Full Scale IQ | | 96 | 96 | 87 | 100 | 94 | 123 | 84 | 91 |
| Verbal IQ | | 103 | 103 | 93 | 99 | 103 | 122 | 90 | 96 |
| Performance IQ | | 91 | 87 | 81 | 102 | 84 | 118 | 78 | 86 |
| NART Full Scale IQ | | 118 | 103 | 96 | 92 | 112 | 116 | 98 | 103 |
| **Executive Functions** | | | | | | | | | |
| Verbal Fluency | | | | | | | | | |
| FAS* | | 30–40th | 40–50th | < 10th | 50–60th | 10–20th | < 10th | < 10th | 30–40th |
| Hayling Test | | Average | Abnormal | Low average | Average | Average | Poor | Impaired | Average |
| Stroop C-W Test* | | 14–16th | 20–24th | < 2nd | 100th | 42nd | < 4th | nt | < 2nd |
| MCST | | Normal | Normal | Normal | Normal | Normal | Normal | Normal | Normal |

*Results presented as percentiles.
Abbreviation key: VOSP = Visual Object and Space Perception battery; GDAT = Graded Difficulty Arithmetic Test; GNT = Graded Naming Test; RMW = Recognition Memory test for Words; RMF = Recognition Memory test for Faces; WMS-III = Wechsler Memory Scale 3rd edition; TEA = Test of Everyday Attention; WAIS-R = Wechsler Adult Intelligence Scale-Revised; IQ = Intelligence Quotient; NART = National Adult Reading Test; FAS = Verbal Fluency FAS; Hayling = Hayling Sentence Completion test; C-W = Stroop Colour-Word task; MCST = Modified Card Sorting Test; sd = standard deviation; No. = number; nt = not tested.

Table 3
Scores on the emotion attribution test (raw scores and t-statistics)

| Patient number | SCA type | Emotion attribution test: Happiness | Sadness | Fear | Anger | Embarrassment |
|---|---|---|---|---|---|---|
| 1 | SCA2 | 14 | 11 | **12*** (−2.387) | **7*** (−2.797) | 8 |
| 2 | SCA2 | **11*** (−3.286) | **7*** (−3.494) | **11*** (−3.439) | **7*** (−2.797) | **6*** (−2.394) |
| 3 | SCA2 | 15 | **9*** (−2.264) | 14 | 12 | 14 |
| 4 | SCA1 | 14 | 12 | 13 | 12 | 8 |
| 5 | SCA1 | 13 | 11 | 13 | 10 | 8 |
| 6 | SCA7 | **8**** (−6.220) | **8*** (−2.879) | 15 | 12 | 14 |
| 7 | SCA7 | nt | nt | nt | nt | nt |
| 8 | SCA7 | 15 | 12 | 14 | 13 | **6*** (−2.394) |
| | Controls mean (sd) | 14.36 (1.0) | 12.68 (1.59) | 14.27 (0.94) | 12.32 (1.86) | 11.14 (2.1) |

All results are presented as raw scores. T-statistics are presented for significant results in brackets.
Abbreviation key: sd = standard deviation; nt = not tested;
The performance of SCA patients was compared with controls using the modified t-test (Crawford & Howell, 1998);
* = $p < 0.05$; ** = $p < 0.001$.

Table 4
Scores on the theory of mind test (raw scores and t-statistics)

| Patient number | SCA type | Theory of mind test | | |
|---|---|---|---|---|
| | | Comprehension | Mental justifications | Physical justification |
| 1 | SCA2 | **13**$^{*}$ (−3.463) | 11 | 2 |
| 2 | SCA2 | 14 | 13 | 1 |
| 3 | SCA2 | 14 | 11 | 3 |
| 4 | SCA1 | 14 | **7**$^{**}$ (−4.740) | **7**$^{**}$ (4.602) |
| 5 | SCA1 | 14 | 13 | 1 |
| 6 | SCA7 | 14 | 10 | 4 |
| 7 | SCA7 | 14 | 12 | 2 |
| 8 | SCA7 | 14 | 11 | 3 |
| | Controls mean (sd) | 13.93 (0.26) | 11.73 (0.96) | 2.2 (1.01) |

All results are presented as raw scores. T-statistics are presented for significant results in brackets.
Abbreviation key: sd = standard deviation; nt = not tested;
The performance of SCA patients was compared with controls using the modified t-test (Crawford and Howell, 1998). $* = p < 0.05$; $** = p < 0.001$.

## 4. Discussion

This study provides a preliminary characterisation of the cognitive and social profiles of a small group of patients with SCA2, SCA1 and SCA7, using an identical battery of cognitive and social cognition tests to the one previously used with SCA3 and SCA6 patients (see [32]). Thus it allows for a powerful comparison between individuals affected by these different hereditary ataxias. In addition, our study provides the first characterisation of social cognitive functions in SCA2, SCA1 and SCA7. Moreover, as far as we are aware, no information regarding the cognitive profile of SCA7 patients has so far been reported in the literature. However, given the relatively small sample size and incomplete imaging data, the results of our study should be regarded as preliminary and considered as a basis for future research.

In all three patient groups, the cognitive domains of calculation and perception were well preserved. The cognitive profile of SCA2 appeared most affected, with frequent impairments in attention, non-verbal intelligence and frontal executive functioning, as well as evidence of naming deficits in one patient. Patients with SCA1 showed a relatively intact profile, consisting mainly of impairments in non-verbal intelligence and attentional functions, although this could be an artefact of their shorter disease duration. Even though the difference in disease duration between the three groups was not statistically significant, SCA1 patients showed no executive dysfunction, which suggests that their pathology has not yet extended to fronto-cerebellar connections and is thus in its early stages [49,75]. SCA7 patients demonstrated only some selective deficits. Only two of the three SCA7 patients showed mild impairment in the non-verbal part of the WAIS-R. This pattern has been previously documented in pure congenital ataxia [83]. Out of four tests sensitive to frontal executive dysfunction, the SCA7 patients achieved below average scores on three. This has been documented in other SCA types and may well be the result of early fronto-ponto-cerebellar degeneration in SCA7 [4, 13,14,57]. Notably, the patient with the longest disease duration presented with some visual memory problems, which may be due to the visual loss characteristic of the later stages of SCA7 [6,36].

On tests of social cognition, difficulties in attributing emotions were evident in patients with SCA2 and SCA7. Interestingly, no emotional processing deficits were found in SCA1 patients. Conversely, a Theory of Mind (ToM) impairment was found only in one SCA1 patient, but there was no evidence of this deficit in any of the SCA2 or SCA7 patients.

We compared the cognitive profiles of SCA2, SCA1 and SCA7 patients with those of our previously reported SCA3 and SCA6 patients, who were tested with the exact same battery [32]. As reported above, SCA1 patients presented with a relatively intact profile, while SCA7 patients demonstrated only some selective deficits. Interestingly, SCA1 patients showed no frontal executive impairments. These findings are comparable with those of SCA6, which was also characterised by a relatively intact cognitive profile. Similarly to SCA7 patients, SCA6 patients also had frontal executive dysfunction. These profiles correspond well with the pathology of SCA1 and SCA7, and the pure cerebellar degeneration of SCA6 [55,59]. We found that patients with SCA2 had the greatest cognitive impairment, which is similar to the pattern which we previously documented in SCA3. This finding coincides well with the more extensive pathology that is associated with these two forms of ataxia [26,27,60]. Overall, the severity of cognitive decline in these patients coincides well with their pathological and clinical appearance.

The cognitive impairments found in our patients replicate the findings of previous studies of some SCA types, which documented deficits in executive functions and attention in SCA2 [14,31,51,85], as well as in attention in SCA1 [13]. Our results also coincide with studies of patients with cerebellar damage, which found deficits in naming [21], attention [87], non-verbal intelligence [83], memory [38,53] and executive functioning [2,39]. Moreover, neuroimaging studies have found activations in the cerebellum in healthy volunteers during tests of attention [8], memory [63] and executive functions [54,66,70]. These deficits are likely the result of damage to cerebro-cerebellar connections [12].

On social cognition tests, patients with SCA2 and SCA7 provided fewer correct attributions of some of five basic emotions (happiness, sadness, fear, anger, and embarrassment) to characters of short stories, compared with controls. This deficit was more prominent in SCA2 patients, probably due to their more extensive pathology. None of the SCA1 patients were impaired on this test. This finding is comparable to the unimpaired performance we previously documented in our SCA3 and SCA6 patients [32]. The deficit found in SCA2 and SCA7 patients corroborates previous findings of cerebellar involvement in emotional processing. Patient studies report an impaired ability to read emotions in the eyes, reduced experience of pleasure, emotional liability, impaired affect regulation, pathological laughter or crying, and unusual brain activation patterns to emotional stimuli [1,53,65,67,89]. Healthy controls show cerebellar activation during emotional processing tasks [33,45,68], whilst stimulation of this structure affects mood and alters task performance [79, 80].

Performance on the ToM task was impaired only in one of two SCA1 patients, but was normal in SCA2 and SCA7 patients. The patient whose performance was impaired provided fewer mental and more physical state justifications than controls for the behaviour of characters in short stories. Interestingly, SCA3 and SCA6 patients were also impaired on this task [32]. The involvement of the cerebellum in ToM has been previously reported both in patients [1,90] and in imaging studies [10,16], as well as in the neurodevelopmental disorder of autism [5,19,73]. MRI investigations in this particular SCA1 patient found no lesions within the posterior fossa. However, it is possible that this patient had atrophy of the vermal lobules specifically implicated in ToM, which may only be detectable with scanning of a higher resolution [93]. Notably, although ToM impairments are often associated with executive dysfunction, this patient performed well on all tests of executive functioning. However, a similar dissociation between executive functioning and ToM has been previously reported in the literature [30].

Therefore, the current study suggests that spinocerebellar ataxias may be characterised by social cognitive deficits. These abnormalities are likely the result of disruptions to connections between the cerebellum and the limbic system, including the amygdala, hippocampus and septum [42,43]. Specifically, there is evidence for direct connections between the vermis, fastigial nucleus and midline folia of the cerebellum and the abovementioned supratentorial limbic regions [43,44]. Our results suggest that there may be some heterogeneity in the pattern of social cognitive impairments in SCA patients, and we propose that this may be attributable to the extent and location of the cerebellar lesion. This would imply that different social cognitive functions are modulated by different cerebellar regions or networks.

Overall, the current study provides a preliminary tentative characterisation of the cognitive and social profiles of patients with SCA2, SCA1 and SCA7, which were directly compared with the profiles of SCA3 and SCA6 patients who were tested previously with an identical battery [32]. We posit that the distinct impairment profiles of the five patient groups can be explained by the segregation of function within the cerebellum, as originally proposed by Schmahmann and Sherman [77] in their description of the CCAS and further developed in more recent studies (e.g. [84]). Importantly, this study provides the first detailed description of the cognitive and social impairments of patients with SCA7. Moreover, the study highlights the possibility that SCA patients may suffer not only from cognitive deficits, but also from social cognitive difficulties- a possibility which should be explored in larger samples with more varied tests. We propose that although there is some homogeneity within each SCA type, there is a degree of heterogeneity in the type of impairments observed across patients, both within and across SCA types.

## Appendix – Social cognition task details

**Emotion Attribution Task:** Examples of the stories used to elicit emotion attributions

*Happiness*: 'Simon has just been told that his work within the company has meant that he will be winning an award.'

*Sadness*: 'Margaret has just found out that her mother has died.'

*Fear*: 'Harriet has woken up and can hear a burglar moving around the house.'

*Anger*: 'Chris is waiting patiently for a car to leave so that he can take their spot. However just as he is about to drive in another car moves into the space.'

*Embarrassment*: 'Neal is driving along the street when he sees one of his friends. He waves but does not see the post box. He drives right into the post box. Lots of people in the street turn around and look at him.'

**Theory of Mind Task:** Example of a theory of mind story

'Today James is going to Clare's house for tea. He is looking forward to seeing Clare's dog, which she talks about all the time. James likes dogs very much. When James arrives at Clare's house, Clare runs to open the door and her dog jumps up to greet James. Clare's dog is huge, it is almost as big as James! When James sees Clare's huge dog he says, 'Clare you haven't got a dog at all. You have got an elephant.'

Comprehension question: Is what James says true?

Theory of mind question: Why does James say this?

Example of a physical interpretation response: "Because the dog is as big as an elephant'.

Example of a mental state interpretation response: 'He is teasing her.'
