
JOURNAL INFORMATION
==============================
NLM Title Abbreviation: Behav Neurol
Journal ID: Behav Neurol
NLM Journal ID: 8914585
Journal ID: BN

Behavioural Neurology

ISSN: 0953-4180
EISSN: 1875-8584
Publisher: Wiley

ARTICLE INFORMATION
==============================
PMCID: PMC5434394
PMID: 28559647
DOI: 10.3233/BEN-2010-0280
Article ID: 261718
Article version: 1
Subjects: Erratum

Preliminary Characterisation of Cognition and Social Cognition in Spinocerebellar Ataxia Types 2, 1, and 7

Sokolovsky N. 1
Cook A. 2
Hunt H. 1
Giunti P. 2
Cipolotti L. 1 3 *
1 Department of Neuropsychology National Hospital for Neurology and Neurosurgery Queen Square London UK
2 Department of Molecular Neuroscience Institute of Neurology UCL Queen Square London UK
3 Dipartimento Di Psicologia Università Degli Studi Di Palermo Palermo Italy
*L. Cipolotti: l.cipolotti@ion.ucl.ac.uk
Print publication date: 2010
Electronic publication date: 2010 Nov 23
Volume: 23
Issue: 3
First page: 159
Last page: 159
Received 2010 Nov 19; Accepted 2010 Nov 19
Copyright: Copyright © 2010 Hindawi Publishing Corporation and the authors.
Copyright year: 2010
License: This is an open access article distributed under the Creative Commons Attribution License, which permits unrestricted use, distribution, and reproduction in any medium, provided the original work is properly cited.
License URL: https://creativecommons.org/licenses/by/3.0/

Erratum for: A Preliminary Characterisation of Cognition and Social Cognition in Spinocerebellar Ataxia Types 2, 1, and 7 23 1-2 16 8 2010 17 29 Behavioural Neurology 10.3233/BEN-2010-0270 PMC5434399 20714058

==============================
Behavioural Neurology 23 (2010) 159
DOI 10.3233/BEN-2010-0280
IOS Press

### Erratum

**A Preliminary Characterisation of Cognition and Social Cognition in Spinocerebellar Ataxia types 2, 1, and 7**

N. Sokolovsky, A. Cook, H. Hunt, P. Giunti and L. Cipolotti

[*Behavioural Neurology* **23**(1,2), 2010, 17–29, DOI10.3233/BEN-2010-0270]

On page 17, in the affiliations and footnote, the second corresponding author P. Giunti was missing. The corresponding author information should be:

Corresponding authors: Prof. Lisa Cipolotti, PhD, Department of Neuropsychology, Box 37, National Hospital for Neurology and Neurosurgery, Queen Square, WC1N 3BG, London, UK. Tel.: +91 0207829 8793; Fax: +91 080 7813 2516; E-mail: l.cipolotti@ion.ucl.ac.uk; P. Giunti, MD, PhD, Department of Molecular Neuroscience, Institute of Neurology, UCL, Queen Square, London, WC1N 3BG, UK. E-mail: p.giunti@ion.ucl.ac.uk.

ISSN 0953-4180/10/$27.50 © 2010 – IOS Press and the authors. All rights reserved
